# Pivotal role of multiphase computed tomography angiography for collateral assessment in patients with acute ischemic stroke

**DOI:** 10.1007/s11547-023-01668-9

**Published:** 2023-06-23

**Authors:** Giorgio Busto, Andrea Morotti, Edoardo Carlesi, Alessandro Fiorenza, Francesca Di Pasquale, Sara Mancini, Ivano Lombardo, Elisa Scola, Davide Gadda, Marco Moretti, Vittorio Miele, Enrico Fainardi

**Affiliations:** 1 Neuroradiology Unit, Department of Radiology, Careggi University Hospital, Florence, Italy; 2 Neurology Unit, Department of Neurological Sciences and Vision, ASST Spedali Civili, Brescia, Italy; 3 Diagnostic Imaging Unit, Department of Experimental and Clinical Biomedical Sciences, University of Florence, Florence, Italy; 4 Department of Radiology, Careggi University Hospital, Florence, Italy; 5 Neuroradiology Unit, Department of Experimental and Clinical Biomedical Sciences, University of Florence, Florence, Italy; 6 Struttura Organizzativa Dipartimentale di Neuroradiologia, Dipartimento di Scienze Biomediche, Sperimentali e Cliniche “Mario Serio”, Università Degli Studi di Firenze, Ospedale Universitario Careggi, Largo Brambilla 3, 50134 Florence, Italy

**Keywords:** Multiphase CT angiography, Collaterals, Acute ischemic stroke, Reperfusion therapies

## Abstract

The cerebral collateral circulation is the main compensatory mechanism that maintains the ischemic penumbra viable, the tissue at risk for infarction that can be saved if blood flow is restored by reperfusion therapies. In clinical practice, the extent of collateral vessels recruited after vessel occlusion can be easily assessed with computed tomography angiography (CTA) using two different techniques: single-phase CTA (sCTA) and multi-phase CTA (mCTA). Both these methodologies have demonstrated a high prognostic predictive value for prognosis due to the strong association between the presence of good collaterals and favorable radiological and clinical outcomes in patients with acute ischemic stroke (AIS). However, mCTA seems to be superior to sCTA in the evaluation of collaterals and a promising tool for identifying AIS patients who can benefit from reperfusion therapies. In particular, it has recently been proposed the use of mCTA eligibility criteria has been recently proposed for the selection of AIS patients suitable for endovascular treatment instead of the current accepted criteria based on CT perfusion. In this review, we analyzed the characteristics, advantages and disadvantages of sCTA and mCTA to better understand their fields of application and the potential of mCTA in becoming the method of choice to assess collateral extent in AIS patients.

## Introduction

The collateral circulation consists of the opening of alternative vascular channels distal to an occluded intracranial artery resulting in a massive vasodilatation that improves blood flow in hypoperfused brain regions. In acute ischemic stroke (AIS), collaterals represent the more important compensatory mechanism maintaining viable the ischemic penumbra, the reversibly damaged brain tissue at risk for infarction surrounding the irreversibly injured infarct core. The ischemic penumbra is the target of reperfusion therapies since it is potentially salvageable [1]. The prognostic value of collaterals in AIS patients is now generally accepted. Poor collaterals are predictors of unsuccessful recanalization and unfavorable clinical outcomes, while robust collaterals are associated with high recanalization rate, tissue reperfusion, early clinical improvement, small Final Infarct Volume (FIV), low risk of hemorrhagic transformation (HT) and favorable clinical outcomes [2, 3]. In addition, collaterals are associated with reduced ischemic core growth and allow the identification of patients showing rapid or delayed infarct expansion (namely fast or slow progressors) [4]. Therefore, collaterals could play a crucial role for the selection of patients candidates for intravenous thrombolysis (IVT) and endovascular treatment (EVT) in both early and late time windows [2]. Different imaging modalities are used for an appropriate evaluation of collateral extent that has become a major need in clinical practice [1, 2, 5, 6]. Digital subtraction angiography (DSA) is still considered as the gold standard for accurate identification of occlusion site and measurement of collaterals due to its high temporal and spatial resolution. In this setting, the American Society of Interventional and Therapeutic Neuroradiology/Society of Interventional Radiology (ASITN/SIR) scale [7, 8] and Careggi Collateral Score (CCS) [8–10] are some of the classifications used for collateral grading with DSA. However, DSA has many limitations since it is an invasive and time-consuming procedure, requires multiple acquisition to visualize both anterior and posterior collaterals and is at risk for thromboembolism. Information on collaterals can also be obtained with perfusion techniques, such as CTP and Magnetic Resonance Perfusion-Weighted Imaging (MR-PWI), using some multiparametric maps and, in particular, the Hypoperfusion Intensity Ratio (HIR) that is calculated on time-to-maximum of the tissue residue function (Tmax) maps as the quotient between the lesion volumes with Tmax > 10 s and > 6 s corresponding to the ratio between infarcted tissue and hypoperfused tissue, respectively. Nevertheless, although a strong correlation between HIR and collateral extent has been demonstrated, HIR does not allow a direct visualization of collaterals. Thus, based on these considerations, collateral assessment is currently performed with computed tomography angiography (CTA) that is a widely available, non-invasive, safe and therefore feasible method [2, 5, 6]. Single-phase CTA (sCTA) is able to identify the point of occlusion and collateral filling, but multi-phase CT angiography (mCTA) was recently proposed, consisting of a three-time points acquisition which provides a more detailed evaluation of collaterals and presents several advantages over sCTA. In fact, mCTA demonstrated a better correlation with FIV and functional outcome, and higher interrater reliability compared to sCTA. Moreover, several studies showed that mCTA is a useful tool for patient selection undergoing EVT, especially in the extended time window where the collateral assessment with mCTA could replace the current approach based on CT perfusion (CTP) parameters [6].

## Functional anatomy of collateral circulation

The cerebral collateral circulation is a vascular system that regulates cerebral blood flow (CBF) in case of vessel occlusion and consists of a network of anastomoses belonging to both the arterial and venous circulation [1, 2, 11–13]. On the arterial side, three principal routes exist: (1) collaterals between the intracranial and the extracranial arteries provided by ophthalmic and superficial temporal arteries; (2) collaterals between the intracranial arteries based on the circle of Willis, a ring-shaped circuit of vessels in which the anterior communicating artery promotes interhemispheric blood flow and the posterior communicating arteries connecting the anterior and posterior circulation; (3) collaterals between the intracranial arteries provided by pial or leptomeningeal branches which generate a communication among the three large vessels of each hemisphere (anterior, middle and posterior cerebral arteries) supplying the cortical surface. At intracranial level, collaterals represented by the components of circle of Willis are indicated as primary, whereas pial (leptomeningeal) collaterals are considered as secondary because are usually recruited when primary collaterals fail. Of note, a variable anatomic configuration is typical of the circle of Willis since the anterior portion results to be complete in only 68%, the posterior portion in 47% and the entire circle in only 36% of individuals. In this regard, it has recently been suggested that the different structures of the circle of Wills could be implicated in defining fast progressors and could correlate with poor collateral score [14]. A high anatomic variability is also characteristic of venous collateral circulation collaterals that increases CBF drainage in case of occlusion of prominent pathways or venous hypertension and allows the exit of blood from the brain through multiple routes. Functionally, collateral activation is modulated by sympathetic system via intrinsic and extrinsic innervations which act on parenchymal vessels and vascular branches on the brain surface, respectively [12]. However, the two key events leading the opening of collaterals are the recruitment and the remodeling [1, 2, 11, 12]. The collateral recruitment can occur early, immediately or in minutes, or can be delayed, as typically occurs in chronic obstructive disease but sometimes appears within a few hours also after acute vessel occlusion providing a potential explanation for spontaneous acute tissue reperfusion. This recruitment is due to the drop of perfusion pressure in vessels downstream the occlusion with formation of a pressure gradient favoring the diversion of blood flow into collaterals and the shear stress that induces vasodilatation. Nevertheless, collateral recruitment is impaired by several conditions including advanced age, chronic hypertension, cerebral small vessel disease [1]. A greater lumen expansion of collaterals associated with increase in tortuosity, vessel length and wall thickness represents the collateral remodeling that develops in days or weeks, mainly during conditions characterized by a chronic decreased in CBF such as stenosis or occlusion of the internal carotid artery.

## CTA acquisition and collateral grading

### sCTA technique

**Fig. 1:**
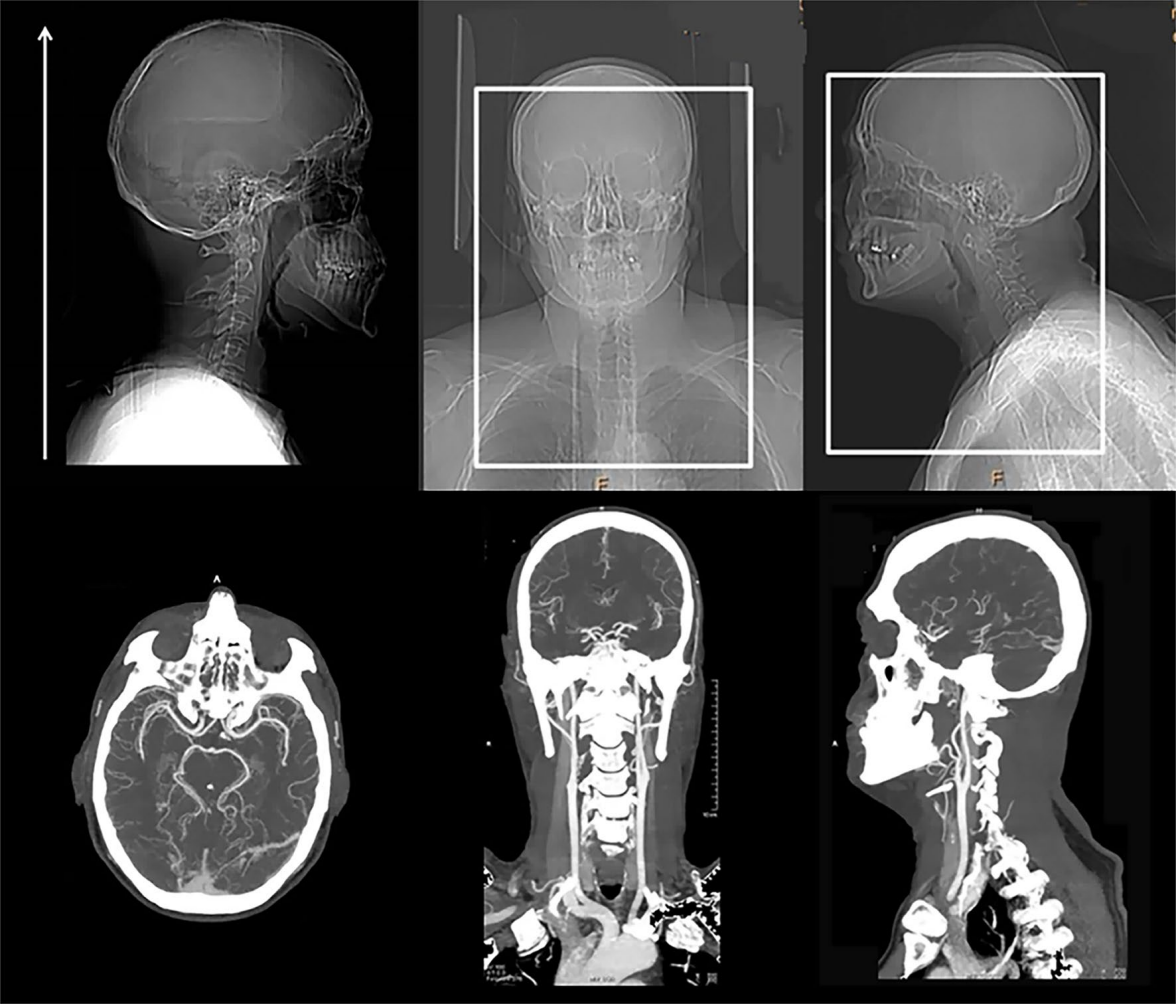
Single-phase CT angiography covers from the aortic arch to vertex and maximum intensity projection reconstructions for the visualization of cervical and intracranial vessels. CTA of the cervical and intracranial vessels was performed as follows: 0.7 mL/kg contrast (maximum 90 mL), 5- to 10-s delay from injection to scanning, 120 kV, 270 mA, 1 s/rotation, 1.25-mm-thick slices, and table speed 3.75 mm/rotation. The axial images were reconstructed at 1-mm overlapping sections, and multiplanar reconstructions for axial, coronal, and sagittal images of the circle of Willis were performed with 3 mm thickness at 1-mm intervals. Thick-section axial maximum intensity projections at 24 mm thickness and 4-mm intervals were also reconstructed

**Fig. 2:**
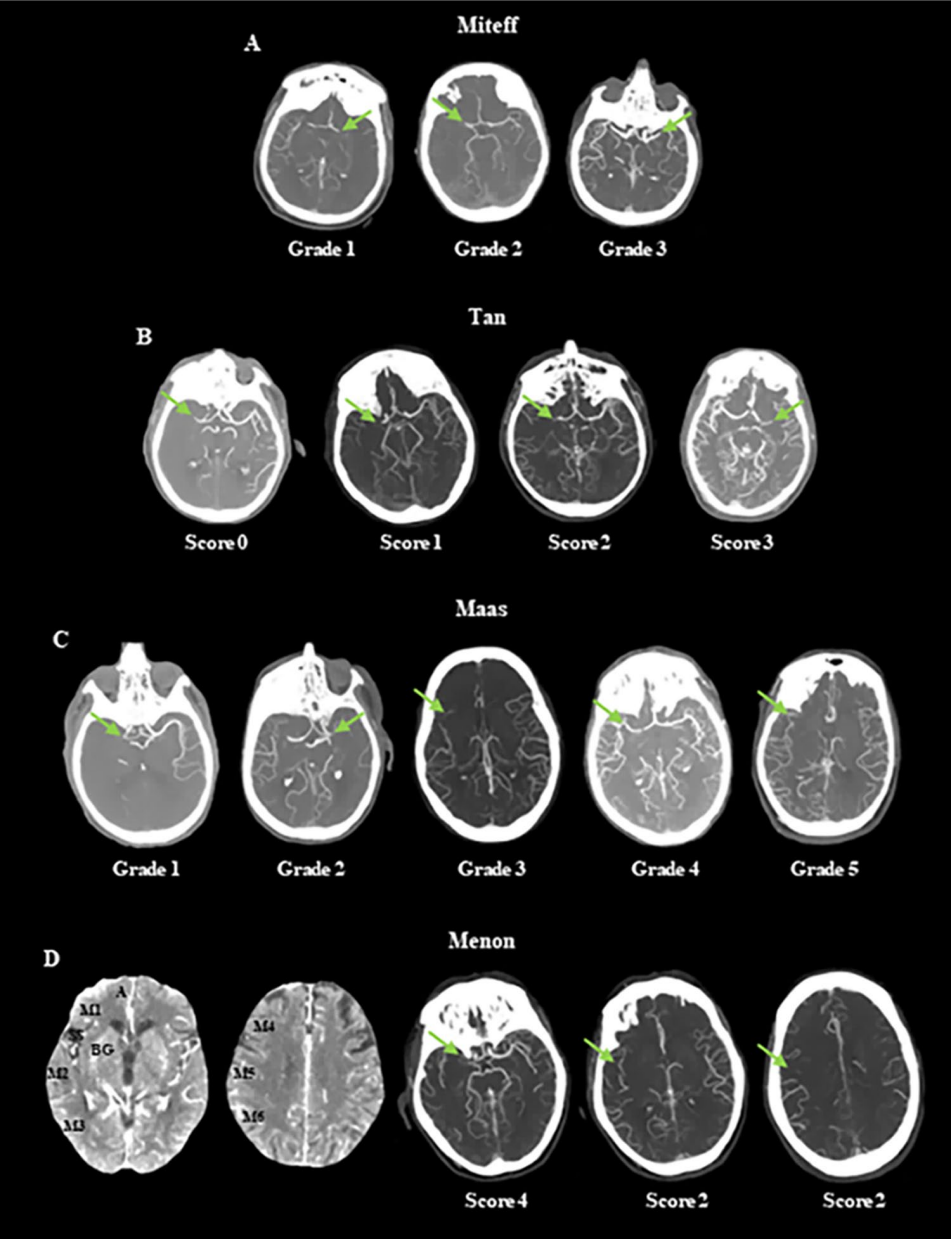
Single-phase CT angiography collateral scores. Green arrows indicate occlusion site or occluded hemisphere. M1, anterior MCA at ganglionic level; M2, lateral MCA at ganglionic level; M3, posterior MCA at ganglionic level; M4, anterior MCA at supraganglionic level; M5, lateral MCA at supraganglionic level; M6, posterior MCA at supraganglionic level; ACA, anterior cerebral artery; BG, basal ganglia

sCTA images are obtained on a standard CT scanner through a volumetric acquisition starting approximately 5–10 s after automatic injection of 50–70 mL of a contrast bolus at the rate of 4–5 mL/s into an antecubital vein. sCTA source images are then reformatted with maximum intensity projection (MIP), a 3D reconstruction algorithm that provides images with a good anatomic detail of cerebral vessels and allows an accurate visualization of collaterals [5, 15]. Usually, sCTA covers from the aortic arch to vertex for the identification of occlusion site at the level of cervical and intracranial vessels (Fig. 1). Several grading scales have been proposed, but four collateral scores based on visual inspection of the degree of collateral filling are the most utilized grading systems in clinical practice (Table 1). The collateral grading system introduced by Miteff and coworkers [16] is a qualitative 3-point scale assigning 3 different grades of retrograde filling to the distal branches of middle cerebral artery (MCA) in the occluded territory that result in poor (grade 1), intermediate (grade 2) and good (grade 3) collaterals (Fig. 2, Panel A). The collateral score suggested by Tan and colleagues [17] is a qualitative a 4-point scale that classifies collateral supply filling of occluded middle cerebral artery territory in 4 different grades corresponding to poor (scores 0–1) and good (scores 2–3) collaterals (Fig. 2, Panel B). The collateral scoring system published by Mass and collaborators [18] is a qualitative 5-point scale that compares the opacification of sylvian and leptomeningeal vessels between occluded territory and contralateral normal side obtaining 5 different grades that represent poor (grade 1), intermediate (grade 2) and good (grades 3–5) collaterals (Fig. 2, Panel C). The collateral score described by Menon et al. [19] is a semiquantitative 20-point score that divides the territories supplied by anterior cerebral artery (ACA) and MCA in 9 regions attributing 0, 2 or 4 points to sylvian scissure and 0, 1 or 2 points to remaining areas after comparison of opacification of pial and lenticulostriate vessels between the occluded territory and contralateral normal side. Collaterals are judged as poor, intermediate and good with a scoring of 0–10 points, 11–16 points and 17–20 points, respectively (Fig. 2, Panel D). In all these collateral scores, except Tan grading, intermediate and good collaterals are considered as good. However, there is currently no consensus on the standard methodology to use for evaluating collateral extent since in prior publications, Menon scale performed better than Miteff score in predicting core and penumbra volumes likely because of its greater ability to estimate the delay rather than the backflow in the affected territory [20].

**Table 1:** Single-phase CT angiography (sCTA) collateral scores

	Grade 1: opacification only of the distal superficial MCA branches
Miteff	Grade 2: opacification of MCA branches within the sylvian fissure
	Grade 3: opacification of entire MCA distal to the occluded segment
	1 = poor collaterals; 2 = intermediate collaterals; 3 = good collaterals
	Grade 1: absent vessel opacification
	Grade 2: less opacification than the contralateral normal side
Maas	Grade 3: equal opacification to those on the contralateral side
	Grade 4: greater opacification than the contralateral normal side
	Grade 5: exuberant opacification than the contralateral normal side
	1 = poor collaterals; 2 = intermediate collaterals; 3–5 = good collaterals
	Score 0: absent collateral supply to the occluded MCA territory
	Score 1: collateral supply filling > 0% but ≤ 50% of the occluded MCA territory
Tan	Score 2: collateral supply filling > 50% but < 100% of the occluded MCA territory
	Score 3: collateral supply filling 100% of the occluded MCA territory
	Scores 0–1 poor collaterals; scores 2–3 = good collaterals
Menon	
M1	
M2	
M3	Score 0: artery not seen compared with a matching region in the opposite hemisphere
M4	Score 1: less prominent filling compared with a matching region in the opposite hemisphere
M5	Score 2: equal or more prominent filling compared with a matching region in the opposite hemisphere
M6	
ACA	
BG	
	Score 0: artery not seen compared with a matching region in the opposite hemisphere
SS	Score 2: artery less seen compared with the opposite sylvian fissure
	Score 4: same or prominent artery filling compared with the opposite sylvian fissure
	Scores 0–10 = poor collaterals; scores 11–16 = intermediate collaterals; scores 17–20 = good collaterals

MCA, middle cerebral artery; M1, anterior MCA at ganglionic level; M2, lateral MCA at ganglionic level; M3, posterior MCA at ganglionic level; M4, anterior MCA at supraganglionic level; M5, lateral MCA at supraganglionic level; M6, posterior MCA at supraganglionic level; ACA, anterior cerebral artery; BG, basal ganglia; SS, sylvian scissure. In the Menon collateral score, the territories supplied by ACA and MCA are divided into 9 regions (M1, M2, M3, M4, M5, M6, ACA, BG and SS). After comparison of opacification of pial and lenticulostriate vessels between occluded territory and contralateral normal side, 0, 1 or 2 points were attributed to M1, M2, M3, M4, M5, M6, ACA, BG regions, whereas 0, 2 or 4 points were assigned to SS region

### mCTA technique

**Fig. 3:**
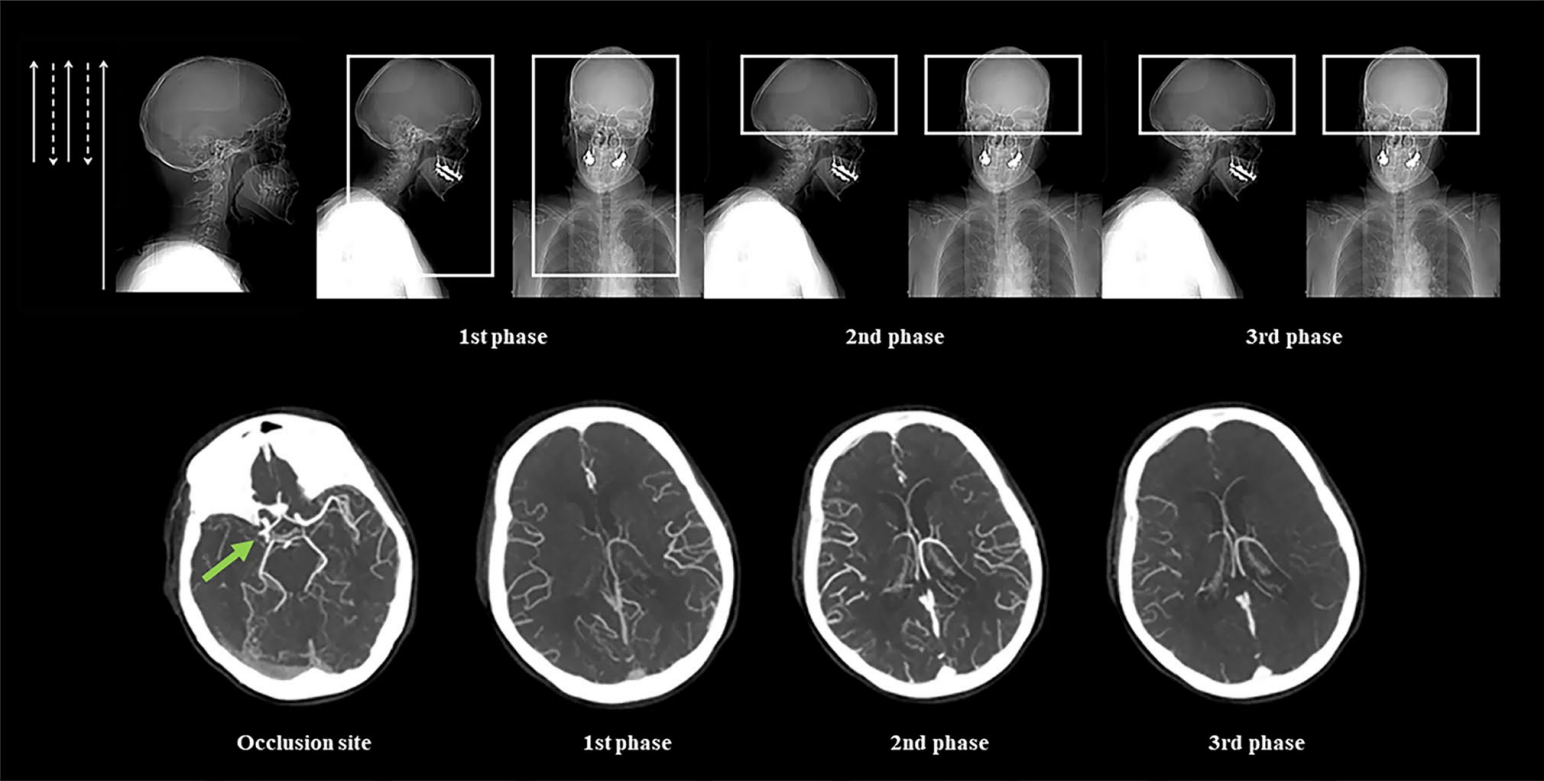
Multiphase CT angiography covering from the carotid bifurcation to vertex (first phase) and from the skull base to vertex (second and third phases) with maximum intensity projection (MIP) reconstructions for the visualization of cervical and intracranial vessels (first phase) and intracranial vessels only (second and third phases). Green arrow indicates occlusion site. CTA of the cervical and intracranial vessels was performed as follows: 0.7 mL/kg contrast (maximum 90 mL), 5- to 10-s delay from injection to scanning, 120 kV, 270 mA, 1 s/rotation, 0.625-mm-thick slices, and table speed 3.75 mm/rotation. The second phase was acquired after a delay of 8 s that allows for table repositioning to the skull base. Scanning duration for each additional phase was 3.4 s. Thus, the three phases were each 12 s apart. The axial images were reconstructed at 1-mm overlapping sections, and multiplanar reconstructions for axial, coronal, and sagittal images of the circle of Willis were performed with 3 mm thickness at 1-mm intervals. Thick-section axial maximum intensity projections at 24 mm thickness and 4-mm intervals were also reconstructed

mCTA is a three-phase acquisition offering time-resolved cerebral angiograms of brain vessels. The first phase replicates the only one phase of sCTA covering from aortic arch to vertex, whereas the following two phases are acquired from the skull base to vertex after table repositioning to the skull base for the visualization of intracranial vessels with a delay 8 s each (Fig. 3). There are no differences in the other characteristics of acquisition compared to sCTA. Therefore, the first phase corresponds to arterial phase, the second phase to equilibrium/venous or peak venous or phase and the third one to late venous phase [21]. mCTA can also be reconstructed from CTP peak of arterial phase obtaining three simulated mCTA phases [22]. In this case, however, the first phase includes only intracranial and not also cervical vessels as in the original mCTA acquisition. Using mCTA, collaterals are assessed with the grading system proposed by Menon and colleagues that is a qualitative 6-point scale (Table 2) in which collateral supply is categorized based on extent and prominence of vascular enhancement after comparison between the occluded territory and contralateral normal side and is identified as poor (grades 0–1), intermediate (grades 2–3) and good (grades 4–5) [21]. In contrast with sCTA classifications, mCTA Menon score defines intermediate profile as poor and not as good collaterals. Of note, while this classification was fully applied in several studies evaluating collaterals with mCTA [23–30], in others collateral filling was measured with sCTA Menon score [17, 20, 29] or sCTA Tan grading [17, 31–33] and intermediate pattern was included in good collaterals. Furthermore, grade 2 instead of grade 4, as stated in the original publication of Menon et al., was found to be the optimal threshold for identifying a good functional independence [27]. Thus, the more accurate score for calculating mCTA collateral extent has to be definitely validated.

**Table 2:** mCTA collateral score from Menon et al.

Grade 0	When compared with the asymptomatic contralateral hemisphere, there are no vessels visible in any phase within the ischemic vascular territory
Grade 1	When compared with the asymptomatic contralateral hemisphere, there are just few vessels visible in any phase within the occluded vascular territory
Grade 2	When compared with the asymptomatic contralateral hemisphere, there is a delay of two phases in filling in of peripheral vessels and decreased prominence and extent or a one-phase delay and some ischemic regions with no vessels
Grade 3	When compared with the asymptomatic contralateral hemisphere, there is a delay of two phases in filling in of peripheral vessels or there is a one-phase delay and significantly reduced number of vessels in the ischemic territory
Grade 4	When compared with the asymptomatic contralateral hemisphere, there is a delay of one phase in filling in of peripheral vessels, but prominence and extent are the same
Grade 5	When compared with the asymptomatic contralateral hemisphere, there is no delay and normal or increased prominence of pial vessels/normal extent within the ischemic territory in the symptomatic hemisphere
	0–1 = poor collaterals; 2–3 = intermediate collaterals; 4–5 = good collaterals

### Automated collateral detection

Several efforts have recently been made to obtain an automated assessment of collateral extent and provide a reliable quantitative collateral scoring, mainly based on artificial intelligence programs. For sCTA, a good agreement was found between visual grading scale proposed by Tan and an automated collateral score performed using in-house [34, 35] or commercial software algorithms, such as Brainomix [36], StrokeViewer [37] and Canon [38]. In addition, a study analyzing patients from MR CLEAN database with in-house software showed that quantitative score was an independent predictor of functional outcome and FIV [34]. For mCTA, the most used automated method is provided by GE Healthcare FastStroke software generating time-variant color-coded maps which correlated well with conventional mCTA collateral score for the evaluation of collateral flow [38, 39], improved the interpretation of collateral status [40] and enhanced the predictive value of collaterals for good outcome [41]. Another in-house software with a good performance for the measurement of collateral supply was more recently described using CTP-derived mCTA images [42]. However, a validation on a larger population of patients is needed before the introduction of these automated software in clinical practice.

## CTA collateral assessment in AIS

A large number of previous studies demonstrated the high predictive value of good collaterals assessed by sCTA for favorable clinical outcome, small FIV and low rates of HT in AIS patients treated with IVT and EVT [43–45]. These findings were confirmed and expanded in a series of recent publications using both sCTA and mCTA techniques, mainly in patients that underwent EVT.

### sCTA collaterals

A clear association between intermediate and good collaterals and favorable clinical outcome was found in a post hoc analysis of Interventional Management of Stroke (IMS) III trial evaluating sCTA collaterals with Maas and Tan scores in AIS patients treated with IVT within 3 h or combined IVT and EVT within 7 h from symptom onset [46]. The same correlation between good collaterals and functional independence was observed in two post hoc analyses of MR CLEAN (Multicenter Randomized Clinical Trial of Endovascular Treatment of Acute Ischemic Stroke in the Netherlands) [47] and DAWN (Triage of Wake-up and Late Presenting Strokes Undergoing Neurointervention With Trevo) [48] trials which measured sCTA collaterals using Tan score in patients receiving EVT at 6 h and at 6–24 h after stroke, respectively. However, benefit from EVT was also seen in patients with poor collaterals rated with Tan scale in an analysis of MR CLEAN Registry [49] and in HERMES (Highly Effective Reperfusion Evaluated in Multiple Endovascular Stroke Trials) meta-analysis including patients who underwent EVT with or without IVT from 4.5 to 12 h after symptom onset [50]. Conversely, in a post hoc analysis of DEFUSE 3 (Endovascular Therapy Following Imaging Evaluation for Ischemic Stroke) no association was reported between sCTA collaterals graded with Maas and Tan scores and clinical outcome in patients treated with EVT at 6–16 h from onset [51]. Next, some investigations showed that good collaterals classified on sCTA with Tan scale predicted successful recanalization after IVT within 4.5 h [52] and EVT up to 24 h [48, 53] after onset, whereas the beneficial effect on clinical outcome of an earlier time to recanalization was independent of collaterals evaluated with Tan score in an analysis of MR CLEAN Registry [54] and detected only in patients with poor collaterals assessed by Miteff scale in a study based on a Korean Registry including patients treated with EVT within 6 h after onset [55]. Nevertheless, in a further analysis of the MR CLEAN Registry successful recanalization after EVT was not affected by sCTA collateral status graded by Tan score [56]. In addition, some studies revealed the predictive value of poor sCTA collaterals scored with Tan and Miteff scales for larger FIV as defined on non-contrast CT (NCCT) or Diffusion-Weighted Imaging (MR-DWI) [48, 57, 58] and increased HT rates [59, 60] in patients treated with EVT up to 24 h from onset. A robust association of good sCTA collaterals with reduced core growth and slow infarct progression was then identified in several studies including untreated patients and patients undergoing EVT within 24 h after onset where Tan [51, 61–64], Maas [65], Menon [66] and Miteff [67] were used as grading scores. In this setting, it is interesting to note that while poor collaterals were associated with penumbral salvage in a population of not treated patients [61, 67], an analysis of Acute Stroke Registry and Analysis of Lausanne (ASTRAL) did not document any correlation between penumbra volume determined by CTP and sCTA collaterals assessed with Tan score in untreated and IVT and/or EVT-treated patients admitted within 24 h from onset, suggesting that the relationship between collaterals and tissue at risk for infarction remains to be completely clarified [68]. Overall, these data indicate that sCTA collateral assessment is an important tool for predicting radiological and clinical outcomes in AIS patients receiving IVT and/or EVT in early and late time windows. On the other hand, sCTA collaterals were also associated with clot characteristics as patients with good collateral score had longer thrombi [69] and elevated thrombus permeability [70], reflecting the presence of residual blood flow through the clot. Finally, a lower edema progression and a greater benefit from EVT in patients with large core have been reported in patients with good sCTA collateral filling [71, 72].

### mCTA collaterals

In the seminal work of Menon and associates the predictive value of mCTA collateral score for functional outcome was modest in AIS patients untreated or treated with IVT and EVT with or without IVT within 12 h of symptom onset [21]. This not excellent association of mCTA collateral assessment with prognosis was confirmed in two recent studies comparing the precision of different grading system applied to mCTA in identifying the degree of functional independence in patients receiving EVT in the same time window. In fact, no significant correlation with clinical outcome was seen for mCTA Menon and sCTA Miteff, Tan and Maas classifications in the first investigation [73], as well as for sCTA Menon and Tan scores in the HERMES analysis [74]. However, the evaluation of collateral filling with mCTA was successfully used as selection tool for AIS patients’ candidates for EVT within 12 h from stroke in the Endovascular Treatment for Small Core and Proximal Occlusion Ischemic Stroke (ESCAPE) trial [31] where patients with good collaterals undergoing EVT achieved more frequently a favorable outcome than controls treated with standard medical therapy. In agreement with these findings, data coming from subsequent studies documented the ability of mCTA scoring system in the prediction of functional outcome. A significant association between good mCTA collaterals and functional independency at 3 months was observed in patients treated within 4.5 after onset with EVT with or without IVT [25] and treated with standard medical therapy, IVT and/or EVT [24]. Next, recent publications showed that patients untreated or treated with EVT and/or IVT within 5 h [75], between 5 and 15 h [22] and within 24 h after ictus [28] who had a good mCTA collateral status at presentation achieved a favorable outcome. In addition, good mCTA collaterals were correlated with small FIV as delineated on NCCT or MR-DWI in patients receiving standard medical therapy, IVT and/or EVT within 4.5 [25], 12 [76, 77] and 24 h of symptom onset [28]. Another association was found between good mCTA collaterals and small admission infarct core as estimated on NCCT Alberta Stroke Program Early Computed Tomography Score (ASPECTS) methodology or MR-DWI in patients untreated or treated with IVT and/or EVT within 8 [26] and 14 h after onset [30]. Finally, good collaterals on mCTA were also linked with slow infarct growth rate in patients treated with IVT and/or EVT within 6 h from symptom onset and achieving successful recanalization [33]. In addition, poor mCTA collaterals were independent markers of malignant infarction defined as development of a large space-occupying brain edema involving at least 2/3 of the MCA territory with ventricles’ compression or midline shift in patients not treated or treated with IVT and/or EVT within 4.5 h from onset [23].

### Superiority of mCTA over sCTA

**Fig. 4:**
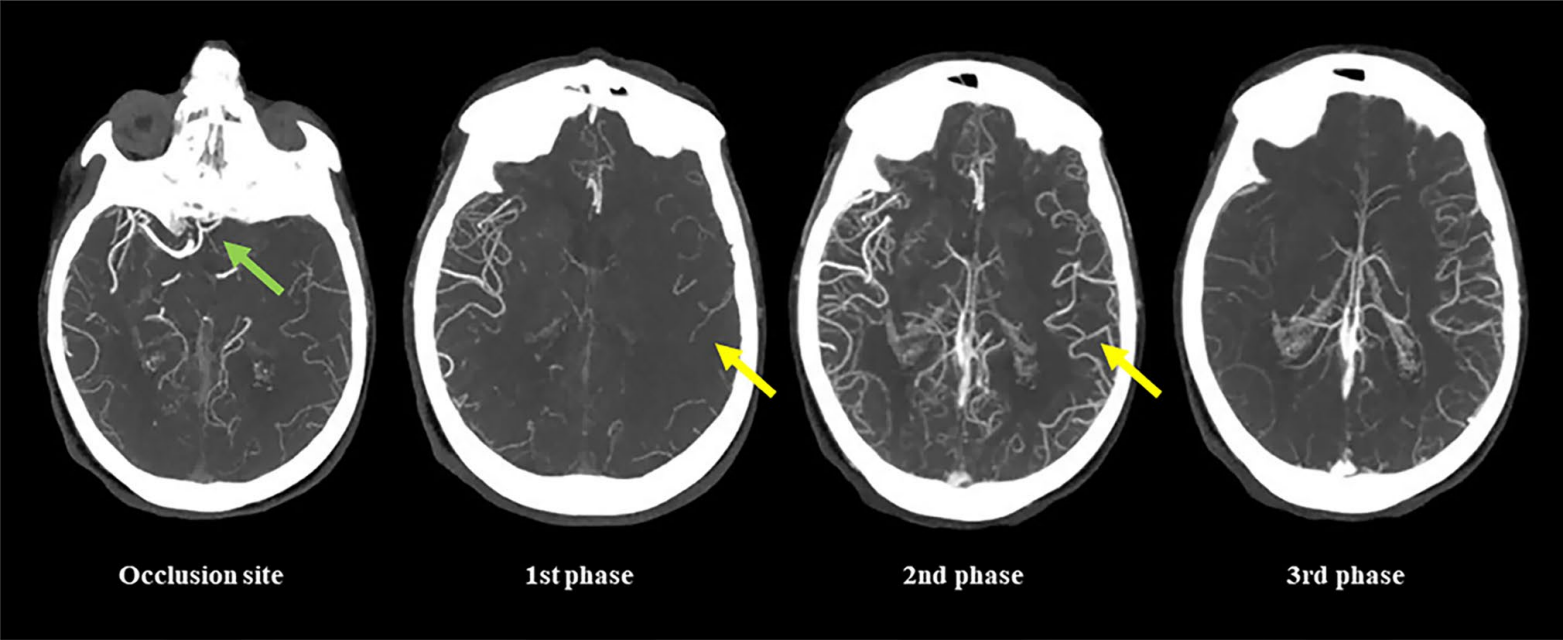
Multiphase CT angiography evaluates collaterals better than single-phase CT angiography. In the first arterial phase corresponding to single-phase CT angiography acquisition collaterals are erroneously judged as poor. On the contrary, the second peak venous phase reveals that collaterals are good. Green arrow shows occlusion site. Yellow arrows indicate collateral extent in the occluded hemisphere

**Fig. 5:**
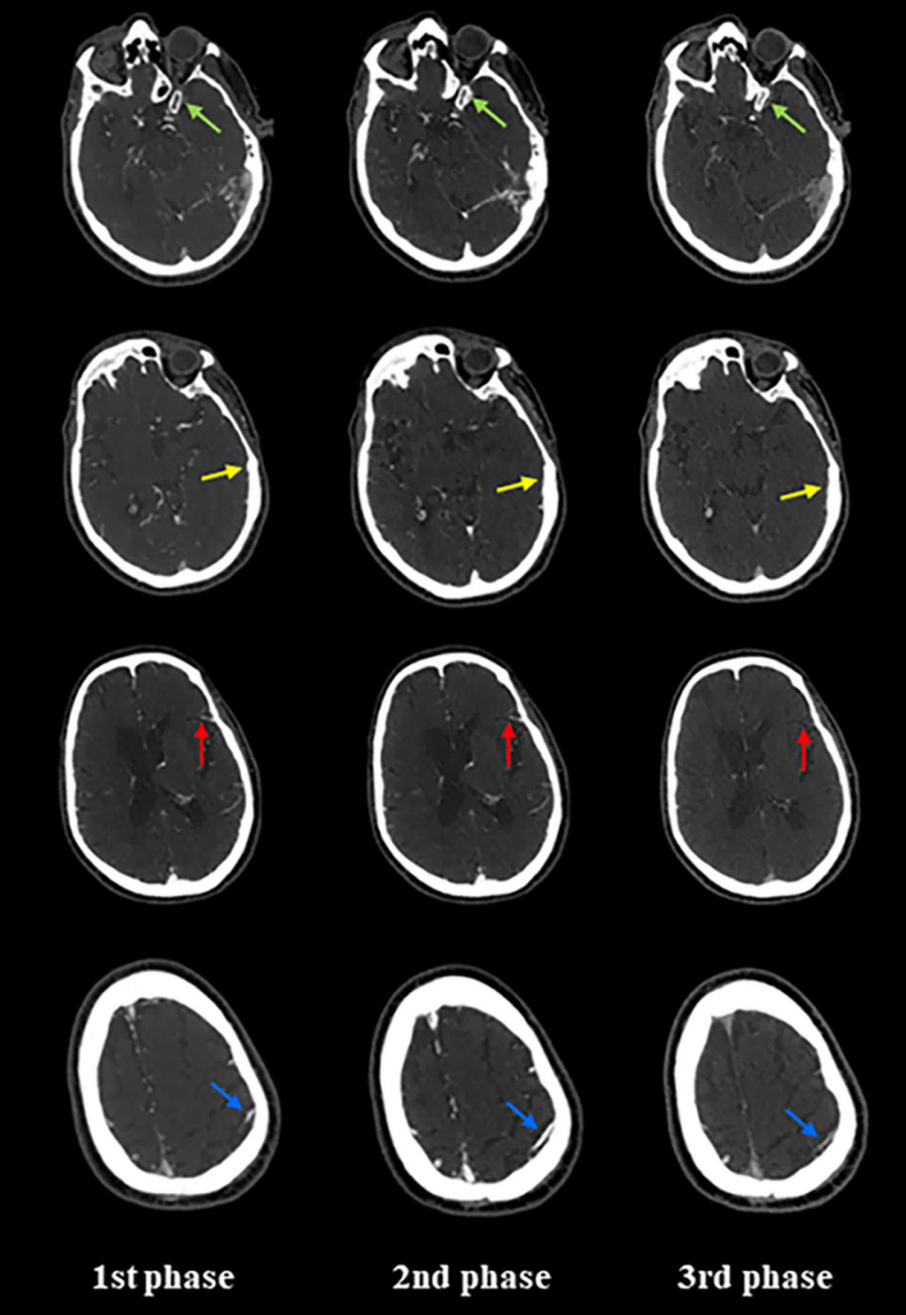
Opacification of sphenoparietal sinus (green arrows), superficial middle cerebral vein (yellow arrows) vein of Labbé (red arrows) and vein of Trolard (blue arrows) within the occluded territory in all three phases of multiphase CT angiography

The main limitation of sCTA is the lack of temporal resolution since the acquisition based on a single time point can lead to an overestimation of collaterals when scans are obtained too late in the venous phase, due to a slow blood flow as a consequence of reduced cardiac output or cervical carotid artery stenosis, or an underestimation of collateral supply when scans are acquired too early in the arterial phase [2, 6, 15]. For the same reason, sCTA is unable to correctly visualize delayed collateral filling during the venous phase with the possibility of considering patients having good collaterals as patients with poor collaterals [6]. These misleading results can be overcome by mCTA that provides three time-resolved images allowing to explore collateral circulation not only in the arterial phase, but also in peak and late venous phases [6, 21]. As a consequence, mCTA ensures a more accurate collateral assessment than sCTA, avoiding misclassification due to the appearance of delayed filling of collaterals in venous phases (Fig. 4). In addition, mCTA shows other advantages over sCTA such as higher interrater reliability and, more important, a superior accuracy in predicting functional outcome at 3 months as demonstrated in the original publication by Menon and associates [21]. Other studies repeatedly confirmed that mCTA is superior to sCTA as prognostic predictor in patients admitted at 4.5–15 h after symptom onset and treated with standard medical therapy and IVT and/or EVT [22, 24, 75]. In this regard, the stronger demonstration that mCTA improves outcome prediction compared to sCTA has been reported by Wang and collaborators [28] where mCTA resulted independently associated with functional outcome in patients untreated and treated with IVT and/or EVT within 24 h after onset, whereas sCTA did not. Intriguingly, mCTA outperforms sCTA also in the analysis of venous outflow at the level of cortical veins representing an indirect indicator of collateral extent and tissue perfusion. In fact, venous drainage reflects the ability of cortical venous system in containing the increased blood volume due to the opening of collaterals and then the effective blood flow traffic through cerebral microcirculation in the ischemic territory [1, 11, 78–80]. sCTA usually determines cortical venous filling according to the Cortical Vein Opacification Score (COVES) proposed by Jansen et al. in a post hoc analysis of MR CLEAN trial that is a qualitative 6-point scale in which the opacification of the superficial middle cerebral vein, the vein of Labbé and the sphenoparietal sinus is classified as complete absence (grade 0), moderate (grade 1) and full (grade 2) after comparison between occluded and unaffected territories. Grades 0–2 indicate poor venous outflow, whereas Grades 3–6 are considered good venous outflow [81]. Several publications reported a strong association of favorable venous outflow with good functional outcome, good collaterals, successful recanalization, lower risk for HT and lower edema progression in AIS patients receiving EVT with or without IVT between 6 and 16 h after symptom onset [80–87]. However, Singh and associates recently analyzed patients treated with IVT and/or EVT within 12 h from onset enrolled in Rapid Assessment of Collaterals Using Multi-Phase CTA in the Triage of Patients with Acute Ischemic Stroke for IV or IA Therapy (PRove-IT) multicenter cohort study [88]. They demonstrated that COVES also including the opacification of the vein of Trolard was more robust as outcome determinant than sCTA COVES. This novel classification, described as total venous score (TVS), is a qualitative 24-point scale in which the filling of the superficial middle cerebral vein, the vein of Labbé, the sphenoparietal sinus and the vein of Trolard is graded as no (grade 0), partial (grade 1) and full (grade 2) opacification in all three phases. The final score is generated by adding the opacification scores calculated in each mCTA phase. In particular, this study documented the relevance of venous opacification during mCTA second and third phases in the actual evaluation of venous filling, suggesting that venous outflow is a time-dependent phenomenon (Fig. 5). Based on these findings, although not without drawbacks as collateral filling could be reduced by flow-limiting proximal stenosis and poor cardiac function [6, 21], mCTA is currently considered the method of choice for collateral assessment in early (0–6 h) and late (6–24 h) time windows for EVT [6].

## Multi-phase CTA collateral-based selection for EVT

DEFUSE 3 [89] and DAWN [90] randomized controlled trials (RCTs) have recently demonstrated the utility of CTP in the identification of AIS patients suitable for EVT in late time window (6–24 h) and patients selected for EVT because of a favorable CTP profile achieved a good outcome more frequently than controls treated with standard care. In both trials, patients were treated if they satisfied specific optimal CTP-derived parameters, collectively named target mismatch, automatically calculated with a dedicated software (RAPID; Rapid Processing of Perfusion and Diffusion; iSchemaView, Menlo Park, CA) using prespecified CTP thresholds. More precisely, absolute time to the peak of the residual function (Tmax) values more than 6 s (Tmax > 6 s) was assumed to indicate critically hypoperfused tissue and relative cerebral blood flow (CBF) values less than 30% of normally perfused tissue (rCBF < 30%) were considered to delineate infarct core. However, despite the successful results of these RCTs, futile recanalization rates were of about 50% [91], suggesting that selection strategy should be improved. ESCAPE trial [31] showed that an mCTA-based selection for EVT could be a promising alternative. Therefore, some research groups have started to explore whether CTA was non-inferior to CTP in the identification of AIS patients who can benefit from EVT. mCTA was initially compared to CTP DEFUSE 3 and DAWN eligibility criteria for the selection of patients’ candidates for reperfusion therapies in two studies evaluating patients treated with EVT or standard medical therapy at 6–12 h after symptom onset from Prove-IT dataset [92] and at 6–24 h after stroke in a Korean single center [93]. In both studies, good collaterals and a good CTP profile were equivalent in predicting favorable outcome. A subsequent study reported that mCTA collaterals were not inferior to perfusion in determining outcome in patients treated with IVT and/or EVT within 24 h of onset [28]. Other two investigations substantially confirmed these findings. Functional outcome was comparable in patients receiving EVT selected with NCCT ASPECTS and mCTA at 6–12 h after onset in ESCAPE Na1 trial [94] and with CTP at 6–16 h and at 6–24 h from onset in DEFUSE 3 [89] and DAWN [90] trial, respectively. More recently, in a Selection Of Late-window Stroke for Thrombectomy by Imaging Collateral Extent (SOLSTICE) Consortium pooled analysis including patients overlapping DEFUSE 3 and DAWN trials as baseline characteristics treated with EVT, the selection with collateral and perfusion imaging showed a similar predictive value for clinical outcome [95]. However, the latest study of Tan et al. did not replicate these results and demonstrated that CTP selection criteria were superior than mCTA eligibility criteria in predicting outcome [96]. Therefore, the possibility that mCTA collateral guided can replace CTP-guided criteria in the selection of patients suitable for EVT in the late time window (6–24 h) still remains matter of debate. In this regard, the relationships between mCTA collateral-based and CTP-based selection criteria were well summarized by Ospel and colleagues which collected data from patients untreated and treated with IVT and/or EVT within 12 h of onset enrolled in Prove-IT study [97]. In this publication, infarct core was defined using three threshold values (rCBF < 30%, CBF < 7 mL/100 g/min, 10 and CBV < 2 mL/100 g) obtaining different results for CTP-guided selection according to the different CTP thresholds utilized. Patients considered eligible for EVT with combined CTP (small core + large penumbra) and mCTA (good collaterals) achieved favorable outcome in 62–87% of cases. In addition, mCTA eligibility criteria selected more patients (91%) than CTP eligibility criteria (7%-36%), but with lower good outcome rates (53–57%). Surprisingly, 51–62% of patients who were not eligible by either mCTA or CTP achieved a good outcome. Therefore, these findings suggest that, although the selection criteria are currently limited, the integration between mCTA collateral-based and CTP-based selection criteria could represent the best paradigm.

## Future perspectives

In line with the observations emerged from the study of Ospel et al. [97], the recently proposed opportunity to generate CTP maps (delay maps) from mCTA could allow to combine mCTA and CTP data for EVT patient selection with the advantage of reducing acquisition time and radiation dose [98, 99]. In the same way, it is now well-accepted that the fate of ischemic tissue depends not only on the amount of blood delivered by collaterals, but also on the blood volume flowing across the microcirculation and drained by venous system. Therefore, the simultaneous assessment of collateral extent by CTA with tissue-level collaterals by HIR and venous outflow by CTA, the so-called cerebral collateral cascade [100, 101], may be a further option for improving our ability to identify AIS patients who actually benefit from reperfusion therapies.
